# Targeting CRABP1 Signalosomes in Managing Neurodegeneration

**DOI:** 10.3390/biom15101428

**Published:** 2025-10-09

**Authors:** Jennifer Nhieu, Li-Na Wei

**Affiliations:** Department of Pharmacology, University of Minnesota Medical School, Minneapolis, MN 55455, USA; nhieu001@umn.edu

**Keywords:** atRA, non-canonical activity, CRABP1, neurodegeneration, AD, PD, HD, ALS

## Abstract

Retinoic acid (RA) binds RA (RAR) and Retinoid X (RXR) receptors to elicit biological effects by regulating transcription. RA is also known to have non-canonical activities mediated, primarily, by cellular retinoic acid-binding protein 1 (CRABP1) which forms protein complexes named “CRABP1 signalosomes” to regulate cytosolic signaling independent of RARs/RXRs. This review focuses on therapeutic applications in neurodegeneration by targeting CRABP1 signalosomes including CRABP1–MAPK, CRABP1–CaMKII, CRABP1–eIF2α, and others recently identified from our proteomic studies. The mouse *Crabp1* gene is regulated by various epigenetic factors and is important for the health of multiple cell types including motor neurons (MNs). In humans, *CRABP1* gene expression is reduced in ALS- and SMA-patient MNs. RA is a therapeutic agent for leukemias and dermatological disorders and is being investigated for managing neurodegenerative diseases, but its therapeutic effects are accompanied by RAR-mediated toxic effects. We have uncovered a novel class of synthetic retinoids that bind CRABP1 without acting on RARs, circumventing RAR-mediated toxic effects. These first-generation CRABP1-selective compounds C3, C4, and C32 target CRABP1–MAPK and/or CRABP1–CaMKII signalosomes. This knowledge, together with emerging structural information, sheds lights on the strategies in designing next-generation CRABP1-signalosome-selective retinoids for the management of neurodegenerative diseases.

## 1. Introduction

Retinoic acid (RA) is the principal active metabolite of vitamin A (retinol) that affects a wide spectrum of biological processes and organ systems such as development, proliferation, differentiation, immunity, vision, cardiac function, and the nervous systems, etc. The pleiotropic effects of RA are primarily mediated by nuclear RA (RAR) and Retinoid X (RXR) receptors that regulate gene transcription. These effects generally span a relatively long period of time, from hours to days, and are irreversible. They are collectively referred to as “genomic” and “canonical” activities. Studies also showed certain non-genomic activities of RA outside the nuclei, reported to be mediated by RARs localized to neuronal dendrites, or by directly binding to protein kinase C to modulate its enzyme activity [1].

RA, especially the most abundant form, all-*trans* RA (atRA), also exhibits rapid specific non-genomic activities in a cell-context-dependent manner, referred to as “non-canonical” activities characterized by (a) RAR/RXR-independence, (b) cytosolic localization, and (c) rapid effects (within minutes) [2,3]. These non-canonical activities of RA have been established to be mediated by a highly conserved, cytosolic RA-binding protein named cellular retinoic acid-binding protein 1 (CRABP1), and have been validated in studies using *Crabp1* gene knockout (CKO) mice and cells. These CRABP1-mediated activities have been demonstrated in multiple organ systems including the heart, the central nervous system (CNS), metabolism, the immune system, and the endocrine system (thyroid gland) [3,4]. Specific to the CNS, CRABP1-mediated activities can affect the maintenance of neural stem cell (NSC) pool (NSC proliferation) [5] and motor activity, specifically in motor neurons (MNs), to maintain the health of neuromuscular junctions (NMJs) [6] and the mitochondrial stress response in MNs [7].

Through molecular studies, it is now known that CRABP1 acts via its ability to form specific protein complexes that modulate cell-context-specific signaling pathways. These CRABP1-containing, signaling-pathway-modulatory protein complexes are named “CRABP1 signalosomes” [3]. Screening libraries of compounds synthesized using a structure-guided design allowed the identification of novel synthetic RA-mimicking compounds, some of which have been validated as signaling-pathway-biased (selective) CRABP1 ligands with specificity/selectivity to particular types of CRABP1 signalosomes [8,9]. This article will review the functions of CRABP1 signalosomes, their physiological implications, and the application of signaling-pathway-biased novel CRABP1 ligands with respect to the management of neurodegeneration. Herein we also propose a novel concept, that it is possible to exploit high-precision signal-modulatory, synthetic CRABP1 ligands (that do not elicit RAR/RXR-mediated retinoid toxicity) to target specific CRABP1 signalosomes and fine-tune signaling pathways that are particularly critical to the health and functions of neurons in a clinical setting, particularly in the management of neurodegeneration.

## 2. Current Therapeutic Applications of Retinoids: An RAR/RXR-Centric Strategy

Retinoids are classified into four generations based on their chemical structure and biological activity towards RARs/RXRs. This classification includes both naturally occurring retinol derivatives and synthetic analogs. RA, particularly atRA, was the first retinoid applied in the clinic, known as tretinoin. Tretinoin is approved by the Food and Drug Administration (FDA) for the treatment of acne vulgaris, photoaging, and a rare form of leukemia known as acute promyelocytic leukemia (APL). In a dermatological context, tretinoin is thought to improve epithelial turnover and regulate sebaceous gland secretion. In APL, tretinoin is thought to induce terminal differentiation of leukemic promyelocytes. The therapeutic efficacy of tretinoin in these clinical applications is primarily mediated by RAR [10,11]. However, it also triggers a wide spectrum of toxicity associated with life-threatening effects including teratogenicity [12] and a severe inflammatory reaction known as differentiation syndrome [13]. To reduce toxicity, subsequent generations of retinoids have been developed with increased selectivity towards specific RAR or RXR isoforms that are thought to elicit distinct effects. However, all the currently FDA-approved retinoids have either warnings, formal contraindications, or a black box warning due to their teratogenic potential [14,15]. Currently approved retinoids and their clinical applications are summarized in Table 1.

In the context of neurodegeneration, RA and synthetic retinoids have also been proposed for the management of neurodegenerative diseases, including Alzheimer’s disease (AD) [16,17,18,19], amyotrophic lateral sclerosis (ALS) [20], Huntington’s disease (HD), [21,22] and Parkinson’s disease (PD) [23,24]. To this end, it is thought that specific RARs or RXRs can be targeted to improve neuronal survival, promote neurogenesis, modulate neuroinflammation, and modulate oxidative stress through antioxidant properties [25]. Other proposed mechanisms include RA’s action as a potential regulator of autophagy and proteostasis [20]. However, all these RAR/RXR-centric strategies cannot avoid widely observed toxicities. Table 2 summarizes retinoids currently under investigation for neurodegenerative diseases.

**Table 1:** Current FDA-Approved Retinoids.

Generic Name	Indications	Mechanism of Action	References
Tretinoin (all-trans retinoic acid, atRA)	Topical Administration: Acne vulgaris Photoaging Oral Administration: Acute promyelocytic leukemia Cystic acne	Pan-RAR (α/β/γ isoforms) agonist	[10,11]
Alitretinoin (9-cis-retinoic acid)	Topical Administration: Kaposi sarcoma skin lesions Oral Administration: Severe chronic hand eczema	Pan-RAR and RXR agonist (α/β/γ isoforms)	[26]
Isotretinoin (13-cis-retinoic acid)	Oral Administration: Severe acne vulgaris	Mechanism not fully understood; proposed to act via isomerization to atRA and 9-cis-RA	[27]
Acitretin	Oral Administration: Severe psoriasis	Mechanism not fully understood; proposed to activate RAR and RXR (α/β/γ isoforms) with higher affinity towards RARs	[28]
Adapalene	Topical Administration: Acne vulgaris	RARβ- and γ-selective agonist	[29]
Tazarotene	Topical Administration: Acne vulgaris Plaque psoriasis	RARβ- and γ-selective agonist	[29]
Bexarotene	Oral Administration: Cutaneous T-cell lymphoma skin lesions	Pan RXR agonist (α/β/γ isoforms)	[30]
Trifarotene	Topical Administration: Acne vulgaris	RARγ-selective agonist	[31]
Palovarotene	Oral Administration: Fibrodysplasia ossificans progressiva	RARγ-selective agonist	[32]

**Table 2:** Retinoids Under Investigation For Neurodegenerative Diseases.

Neurogenerative Disease	Candidate Retinoids	References
AD	atRA Tamibarotene (Am80) AM580 *Bexarotene *HX630	[16,17,18,19,25]
ALS	atRA Adapalene LE-135 Ellorarxine (DC645 or NVG0645) *Bexarotene	[20,25,33]
HD	AC261066 *Bexarotene	[21,22]
PD	Retinol atRA 9-cis RA *IRX4204 *BRF110 *LG268	[22,23,24,25,34]

* Selective towards RXRs; AD—Alzheimer’s disease; ALS—amyotrophic lateral sclerosis; HD—Huntington’s disease; PD—Parkinson’s disease.

## 3. CRABP1 Signalosomes: A Recently Established Principle of atRA Signaling in the Cytoplasm 

**Figure 1:**
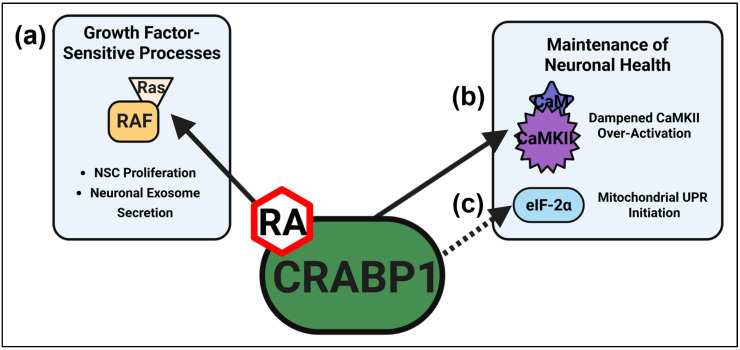
Functions of CRABP1 Signalosomes. (**a**) RA-bound CRABP1 (RA–CRABP1) forms a signalosome with Raf kinase to ultimately regulate the growth-factor-sensitive processes of NSC proliferation and neuronal exosome secretion. (**b**) RA–CRABP1 forms a signalosome with, and dampens the over-activation of, CaMKII. (**c**) CRABP1 can also form a signalosome with eIF-2α to promote initiation of the mitochondrial unfolded protein response (UPR). The dashed line indicates that the CRABP1–eIF-2α signalosome remains to be validated. These CRABP1 signalosomes act to modulate neurons’ proliferation, health, and functions. Image generated using Biorender.com.

CRABP1 was recently identified as the primary mediator of atRA’s non-canonical activity, defined by its ability to rapidly modulate cytosolic signaling in an RAR/RXR-independent manner [2,3]. CRABP1 is a highly conserved protein (>99% conservation across vertebrate species) and was initially characterized merely as a cytosolic binding for atRA with a high affinity. It was originally proposed to sequester and regulate intracellular RA concentrations, presumably involving cytochrome P450 (CYP450) enzymes for catabolism [35,36]. However, recent genetic and molecular studies have uncovered and established that, in fact. CRABP1 mediates the recently reported non-canonical activities of RA by interacting with specific partners to form atRA-stimulated (enhanced) protein complexes. These CRABP1-interacting protein partners are mostly kinases which regulate specific cellular signaling pathways in a cell type/context-dependent manner. These CRABP1 protein complexes are therefore referred to as CRABP1 signalosomes, which have been established to modulate various biological processes/events. These conclusions are based on studies of *Crabp1* gene knockout (CKO) cells and mouse models. Currently, two CRABP1 signalosomes have been established that modulate the health and function of neurons: the CRABP1-mitogen-activated protein kinase (MAPK) and CRABP1-calcium (Ca^2+^)/calmodulin-dependent protein kinase II (CaMKII) signalosomes. Recent proteomic studies further identified 86 additional CRABP1-containing protein complexes, several of which are also putative CRABP1 signalosomes [37]. This section will discuss two established CRABP1–MAPK and CRABP1–CaMKII signalosomes, a recently identified CRABP1–eukaryotic initiation factor 2 alpha (eIF2α) signalosome, and other potential signalosomes that may affect neurons, summarized in Figure 1. Synthetic CRABP1-selecitve ligands modulating CRABP1–MAPK and CRABP1–CaMKII signalosomes are discussed in Section 5.

### 3.1. CRAPB1-MAPK Signalosome in Neurogenesis and Neuronal Exosome Secretion

Molecular studies have established that atRA binding to CRABP1 (RA–CRABP1) enhances CRABP1’s ability to form a protein complex with rapidly accelerated fibrosarcoma 1 (Raf-1), activating downstream kinases mitogen-activated protein kinase kinase 1 and 2 (MEK1/2) and then extracellular-regulated kinase 1 and 2 (ERK1/2) in the MAPK pathway [2]. CRABP1–MAPK signalosome acts to modulate the potency of MAPK signaling, especially in maintaining the stem cell fate of NSCs. Using the CKO mouse model, it has been validated that the CRABP1–MAPK signalosome affects NSC proliferation, particularly in the sub-granular zone of the hippocampus [5]. Mechanistically, CRABP1 competes with rat sarcoma virus (Ras) for Raf-1 binding [2], thereby negatively regulating mitogen-stimulated stem cell proliferation. Therefore, CRABP1–MAPK signalosome activation ultimately slows down NSC cell cycle progression in the presence of growth factors, presumably to maintain a proper NSC pool and to control neuronal differentiation. NSC transplantation has been proposed in managing certain neurodegenerative disorders such as AD, PD, and HD [38]. This aims to supplement NSCs, and maintaining “stemness” is critical. To this end, exploiting CRABP1 ligands specific to the MAPK signaling pathway may be beneficial in maintaining NSC stemness during in vitro manipulation, as well as maintaining the NSC pool in vivo.

The CRABP1–MAPK signalosome was also observed to negatively regulate the secretion of a subset of neuronal exosomes containing a pro-inflammatory protein cargo, receptor-interacting protein 140 (RIP140), at the neuro–immune interface. CKO mice showed elevated levels of pro-inflammatory exosomes in serum and cerebral spinal fluid, reflecting elevated systemic and CNS inflammation. Neuroinflammation is often implicated in neurodegenerative diseases. Several studies have also reported that secreted exosomes from diseased neurons can be taken up by immune cells to promote neuroinflammation [39]. Furthermore, growth factors that activate the MAPK pathway have been associated with an increase in exosome secretion. Therefore, targeting the CRABP1–MAPK signalosome may provide a means to selectively reduce the secretion of pro-inflammatory exosomes, potentially mitigating neuroinflammation in certain neurodegenerative diseases.

### 3.2. The CRABP1–CaMKII Signalosome in Maintaining NMJ Health and Dampening Excitotoxicity in MNs

Molecular studies have revealed that CRABP1 can directly interact with CaMKII to dampen its activation. Mechanistically, CRABP1 competes with calmodulin, thereby dampening CaMKII activation [3]. Our CKO mouse studies first revealed that CRABP1–CaMKII signalosome was protective for cardiomyocytes [3] by dampening CaMKII activation, and later it was also found to be protective for MNs [6,9]. The protective effect of CRABP1–CaMKII is attributed to its ability to dampen aberrant activation (hyper-activation) of CaMKII, which is a major mediator of Ca^2+^ signaling. Ca^2+^ overload and subsequent hyper-activation of CaMKII is implicated in the toxicity and over-excitation of excitable cells including neurons [40,41,42]. In particular, Ca^2+^ overload is a well-known feature of excitotoxicity, which is a major driver of neurodegeneration [41,42,43,44,45].

CRABP1 is highly and specifically expressed in MNs [6], and the protective role of the CRABP1–CaMKII signalosome has been validated in studies of CKO MNs and mouse models. CKO mice spontaneously develop motor dysfunction with apparent morphological defects in, and reduced numbers of, NMJs accompanied by MN degeneration/death. Viral rescue of *Crapb1* expression in CKO MNs can improve motor function, promote neurite outgrowth, and increase NMJ numbers [6]. Therefore, targeting CRABP1–CaMKII signalosome provides an attractive strategy in managing neurological diseases, such as ALS, where aberrant Ca^2+^ signaling/excitotoxicity can be especially detrimental to vulnerable neuron populations such as MNs [43,45]. Additionally, targeting CaMKII can also affect specific neuronal functions such as long-term potentiation (LTP) [46] and thus could also be an attractive target in managing AD [47,48].

### 3.3. The Putative CRABP1–eiF2α Signalosome in Modulating Neuronal Stress Responses

Our recent study showed that CKO MNs have a reduced capacity to execute the mitochondrial unfolded protein response [7]. This stress response involves a series of molecular processes induced by various extra- and intracellular insults such as nutrient deprivation, oxidative stress, and unfolded protein accumulation. A major arm of this response is known as the integrated stress response (ISR) which is activated by the phosphorylation of eIF2α through multiple eIF2α kinases-general control nonderepressible 2 (GCN2), heme-regulated inhibitor (HRI), and protein kinase R (PKR)-like endoplasmic reticulum kinase (PERK) [49]. In CKO MNs, eIF2α phosphorylation is reduced as compared to that in wild-type (WT) MNs, suggesting a failure of CKO MNs to engage the ISR [7]. While it is unclear how CRABP1 modulates eIF2α phosphorylation, our proteomic and molecular studies have revealed that CRABP1 complexes with eIF2α, suggesting the formation of a CRABP1–eIF2α signalosome [37].

Cellular stress is a major contributor to neurodegeneration [50,51,52,53]. The mitochondrial stress response is particularly important in managing oxidative stress by inducing various antioxidant pathways [52,53]. Failure to properly engage ISR can leave the cell vulnerable to various insults. Interestingly, increased eIF2α phosphorylation has also been observed in brain tissues of AD patients [54]. Improper resolution of the stress response, marked by prolonged eIF2α activation, can result in cell death. Therefore, targeting the CRABP1–eIF2α signalosome may be beneficial to the management of stress-induced neurodegenerative processes.

### 3.4. Other Potential CRABP1 Signalosomes in Neurodegeneration

Neurodegeneration is a multifaceted process that includes accumulation of disease-associated proteins, dysregulation of signaling, structural defects, failure to respond to insults, and many more [55]; therefore, multiple approaches may be needed to manage neurodegeneration. Our recent proteomics studies utilizing immunoprecipitation-mass spectrometry (IP-MS) have revealed 86 additional proteins that can form complexes with CRABP1, and therefore, they are also potential CRABP1 signalosome components. Bioinformatic analyses have revealed that several of these proteins can be related to distinct molecular functions represented by gene ontology (GO) terms [37]. The top five GO terms ranked by the highest number of associated IP-MS proteins are as follows: actin filament binding, RNA binding, ubiquitin-protein transferase regulator activity, protein kinase binding, and GTPase activity. Dysregulation or aberrant alterations in these processes have been implicated in various neurodegenerative diseases, providing avenues for the development of therapeutics that target CRABP1 signalosomes. Table 3 summarizes findings related to these molecular functions and their associated IP-MS proteins in neuronal processes implicated in neurodegenerative diseases.

## 4. Physiological Considerations: Regulation of CRABP1 Expression in Neurons

Given that CRABP1 acts by forming cell-context-dependent complexes with various kinases to modulate certain signaling pathways in specific cell types, the abundance of CRABP1 expression in those cells would be critical to its biological activities. Therefore, the expression level of CRABP1 must be tightly and cell-context-dependently regulated in various cell types. In fact, using mouse and cell-culture models, earlier studies have shown tight regulation of the mouse *Crabp1* gene, including its regulation by RA, thyroid hormones, and specific epigenetic factors such as DNA methylation and certain chromatin remodeling machinery [90,91]. Evidence has also been obtained showing that appropriate induction of *CRABP1* expression is critical for the development and maintenance of MNs [92].

On the translational front, human studies have revealed single-nucleotide polymorphisms (SNPs), particularly within the human *CRABP1* gene promoter region, in ALS patients [3]. Presumably, genetic variations affecting *CRABP1* gene promoter activity in MNs can alter CRABP1 expression and contribute to the health and function of MNs, as well as their vulnerability to stress. Analyses of tissues from individuals with MN diseases, including ALS [93,94] and SMA [95], consistently show down-regulation of CRABP1 expression in affected neurons. These findings support the notion that maintaining a sufficient and proper amount of CRABP1 is essential for maintaining neuronal health and function by preserving the protective and modulatory capacity of CRABP1 signalosomes. As such, it may also be clinically relevant to understand how disease-associated promoter variants disrupt the regulatory mechanisms of the *CRABP1* gene, which may shed light on certain specific disease etiologies. This can provide valuable insights into novel therapeutic strategies, such as restoring the capacity of specific CRABP1 signalosomes in managing neurodegeneration by inducing proper *CRABP1* expression.

## 5. A New Class of Retinoids: CRABP1-Dependent Signal Modulators

As explained in previous sections, specific CRABP1-binding synthetic retinoids that elicit activities similar to the non-canonical activities of atRA can be designed. These represent a new class of synthetic retinoids that specifically target CRABP1 without engaging RARs/RXRs; therefore, they are not likely to induce typical retinoid toxicities. By screening libraries of synthetic retinoids, we have identified and characterized a new class of synthetic retinoids characterized according to the following criteria: (1) CRABP1-binding, (2) no activation of RAR, and (3) ability to modulate CRABP1 signalosomes. In the following sections, the characteristics of this new class of synthetic retinoids and their potential applications are discussed.

### 5.1. First-Generation CRABP1-Signalosome-Targeting Synthetic Retinoids

**Figure 2:**
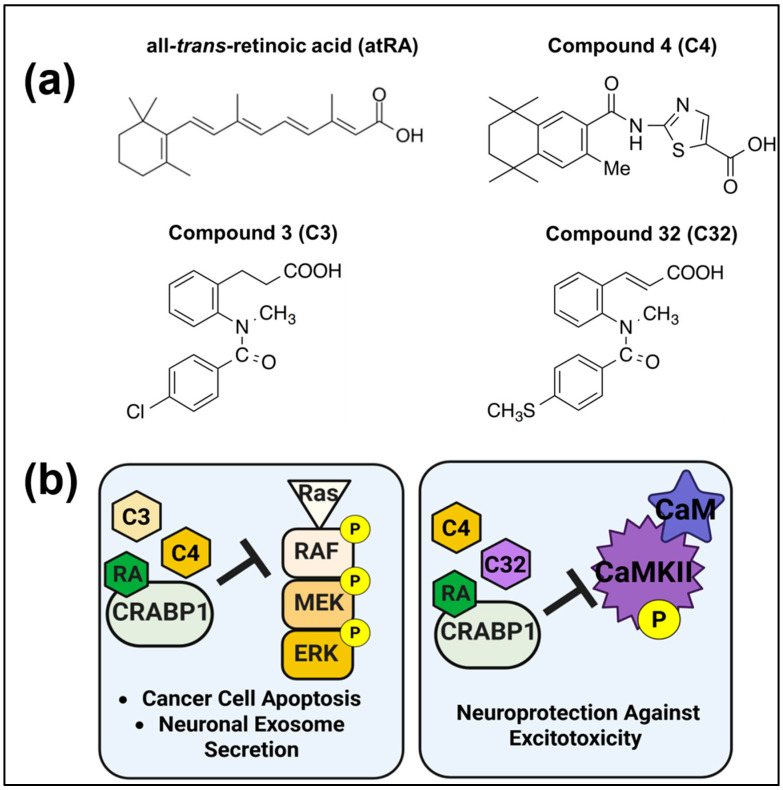
First-Generation CRABP1-Signalosome Modulators. (**a**) Chemical structures for atRA and CRABP1 ligands C3, C4, and C32. (**b**) Activities of CRABP1 ligands. Left: RA, C3, and C4 dampen growth-factor-mediated MAPK activity to induce cancer-cell apoptosis and regulate neuronal exosome secretion. Right: RA, C4, and C32 dampen CaMKII over-activation to protect against excitotoxic insult in motor neurons. Image generated using Biorender.com.

To date, we have identified three compounds, C3, C4, and C32, that exhibit the characteristics of CRABP1-signalosome modulators (Figure 2a,b). These compounds were designed based on the known structure model of atRA-bound CRABP1 [96]. Additionally, C3, C4, and C32 were observed to compete with atRA for binding to CRABP1, indicating that these compounds likely also bind within the atRA-binding pocket of CRAPB1 [8]. C3 and C4 were initially found to target the CRABP1–MAPK signalosome to induce cancer-cell apoptosis. Specifically, without growth-factor-activated Ras, these compounds could bind CRABP1 to activate ERK1/2 to target protein phosphatase 2 (PP2A), ultimately inducing apoptosis in cancer cells. It was further shown that C3-bound-CRABP1, like RA–CRABP1, could compete with Ras, the upstream activator of Raf-1, for binding to Raf-1 (thus activating ERK1/2). This would indicate that, in the presence of growth-factor-activated Ras, the C3–CRABP1–Raf signalosome could inhibit robust, growth-factor-activated Raf-1 activity, ultimately dampening growth-factor-stimulated ERK1/2 activation in cell proliferation. Additionally, C3 and C4 were also found to dampen growth-factor-stimulated secretion of pro-inflammatory exosomes from cultured hippocampal neurons. These results suggest that, besides modulating NSC cell cycle progression, retinoids targeting CRABP1–MAPK signalosomes may also be useful in modulating inflammation, which is implicated in neurodegenerative disorders.

Subsequently, we found that both C4 and C32, but not C3, can dampen CaMKII activation, mediated by CRABP1. These CRABP1–CaMKII-signalosome-targeting compounds are most useful in protecting MNs from excitotoxic insult. Interestingly, these studies demonstrated that compounds such as C4 can elicit “pan-modulatory” activity because it can target both CRABP1–MAPK and CRABP1–CaMKII signalosomes, whereas C3-type compounds are more selective or biased towards the MAPK signalosome. Whether C32, which is known to target the CRABP1–CaMKII signalosome, can also target the CRABP1–MAPK signalosome remains to be determined. Nevertheless, these results support an emerging concept of designing CRABP1-signalosome-targeting synthetic retinoids to selectively modulate a specific signaling pathway in clinical applications. Presumably, these types of specific CRABP1-signalosome-biased compounds will deliver more efficacious and less toxic therapeutic activities.

### 5.2. A Structural Basis for Designing CRABP1-Signalosome Modulators

CRABP1 is structurally arranged as a 10-stranded, anti-parallel β-barrel, with a helix-turn-helix motif positioned at the entrance of its hydrophobic pocket for ligand binding [97]. Structural studies have established that atRA enters this pocket by first interacting with the helix-turn-helix motif, which guides atRA into the β-barrel’s hydrophobic cavity. Two CRABP1 residues, Arg131 and Tyr133, each make direct contact with atRA; furthermore, mutagenesis studies have established that Arg131 is essential for high-affinity binding [90,91].

A synthetic retinoid developed by Tomlinson et al. (2021) [98], DC645, was found to occupy the expected CRABP1 pocket. Notably, crystal structures revealed that DC645 binding induces positional shifts in several solvent-exposed side chains on the β-barrel surface as compared to apo-CRABP1 [98]. These shifts occur at sites distant from the ligand-binding pocket, indicating that ligand binding can trigger allosteric conformational changes outside of the RA-binding pocket. Interestingly, additional endogenous and synthetic retinoids are also known to bind CRABP1 [99]. It is tempting to speculate that ligands that engage the RA-binding pocket could potentially induce certain allosteric changes. Importantly, this finding by Tomlinson et al. (2021) [98] provides important information consistent with our molecular and structural studies of CRABP1-signalsome interactions. Specifically, we found that CRABP1’s direct interactions with Raf-1 kinase and CaMKII also involve its solvent-exposed β-barrel surface residues [2,3,100], and that both of these interactions are enhanced by atRA binding [3]. Furthermore, mutagenesis studies of the CRABP1–CaMKII signalosome revealed that certain β-barrel residues on CRABP1 determine its interaction preference for inactive versus active CaMKII, providing a mechanistic insight for CRABP1’s ability to dampen CaMKII activation [100]. Therefore, synthetic compounds could be rationally designed not only to bind the CRABP1 pocket but also to induce certain specific allosteric conformations that are important for its specificity/selectivity for particular interaction partners. Such signaling-pathway-specific modulation would enable the design of CRABP1-dependent compounds that selectively activate specific CRABP1 signalosomes, providing a more specific and effective therapeutic strategy that minimizes drug toxicity. The concept of signaling-pathway-specificity (or bias) has been applied in the development of therapeutics to achieve specifically desired biological and therapeutic outcomes. This is exemplified by intense investigation of biased G-protein coupled receptor (GPCR) signaling [101] and selective receptor modulators of nuclear receptors [102]. Given the extremely conserved primary sequence (and thus molecular structure) of CRABP1, its ability to interact with multiple signaling proteins, and its distinct effects on the downstream signaling in various cellular backgrounds, we therefore propose that CRABP1 ligands could play a role in modulating various signal outputs that are critical to certain specific biological processes. To achieve this, CRABP1 would act like a signaling scaffold protein to elicit specific ligand-dependent modulations of various CRABP1–protein complexes.

**Figure 3:**
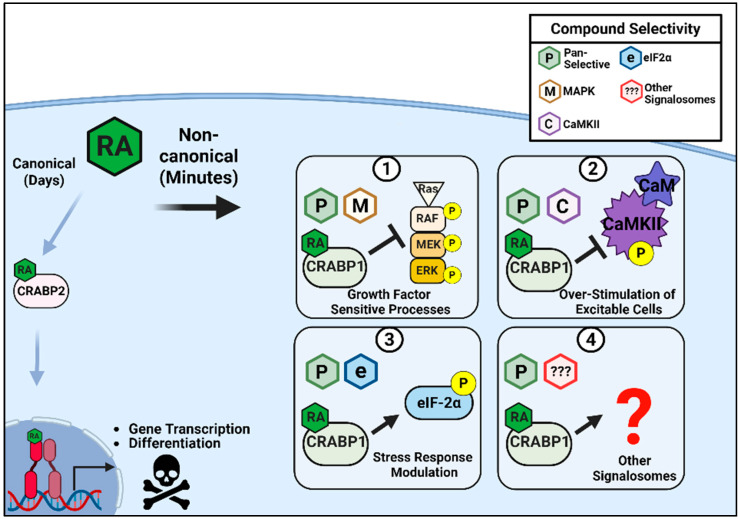
Potential Activities of CRABP1-Signalosome Modulators. RA through RAR-mediated, canonical activities regulate gene transcription as a primary therapeutic mechanism. But this activity is also associated with toxic side-effects. Non-canonical activity of RA through CRABP1 can be targeted with CRABP1-selective ligands to circumvent RAR-mediated toxicities. These ligands are proposed to exhibit the following actions: Pan-selective for several signalosomes (1–4), selectivity towards MAPK in growth-factor-sensitive processes (1), CaMKII in protection against over-stimulation (2), eIF2α in the modulation of the stress response (3), or other currently unknown signalosomes (red question mark) and their biological processes (4). Image generated using Biorender.com.

To this end, exploiting the structure–activity relationship (SAR) and biological-pathway specificity in designing CRABP1 ligands is of great interest in future studies. Figure 3 depicts the range of activities that would be desirable for these novel signalosome modulators that are less likely to elicit RAR/RXR-associated toxicities. We propose that such compounds could be rationally designed to exhibit pleiotropic activities, either through targeting multiple distinct signalosomes as a pan-modulator (Figure 3, boxes 1–4) or by acting more selectively. For example, they could modulate growth-factor–mediated pathways (Figure 3, box 1), reduce hyperexcitability in excitable cell types (Figure 3, box 2), or influence the UPR and other related stress-response pathways (Figure 3, box 3). Additional CRABP1 signalosomes and cellular processes, once elucidated, could also provide information for new therapeutic targets (Figure 3, box 4).

## 6. Future Directions

Given the well-understood structural basis of the CRABP1–ligand interaction, it is a considerably easy task to improve the specificity of compounds for CRABP1 binding. However, the information about how CRABP1 interacts with its binding partners to form a specific CRABP1 signalosome has just begun to be gathered recently. This represents an important area of future research to improve the “signaling-pathway-specificity” of these types of retinoid therapies. Presumably, more powerful techniques detecting specific CRABP1-signalosome-complex formation and its disassembly are needed to improve the design of these types of retinoids. This will facilitate the design of next-generation CRABP1-signalosome-targeting compounds with optimal signaling-pathway selectivity.

Thirdly, identifying and validating additional CRABP1 signalosomes that are physiologically relevant is also an important research area in future studies. In particular, it remains unclear whether there is a neuronal type-specificity of CRABP1 signalosomes. CRABP1 is expressed in certain neurons, such as MNs [6,92] and hippocampal neurons [5]. While it is known to be also expressed in the cortex, it is unclear what type of cortical neurons are CRABP1-positive. Future studies of single-cell expression profile are needed to characterize CRABP1-posive neurons, which will expand the capacity of CRABP1-signalosome-based therapeutic development, especially for various neurodegenerative conditions.

Finally, by incorporating gene/protein delivery for ectopic and transient expression of CRABP1 together with the delivery of CRABP1 ligands, it may also be possible to develop targeted/tailored combination therapies. This approach will also restore CRABP1 levels in certain diseased neurons where CRABP1 is pathologically down-regulated. Given the incidences of disease-associated down-regulation of CRABP1, such as in certain types of ALS, this approach may maximize the potency of precision therapy for those diseases that typically require long-term therapy.

## 7. Conclusions

The establishment of “non-canonical” activity of atRA via CRABP1 challenges the classical, nucleus-centered theory of the activities of vitamin A and provides the basis for the development of novel retinoid-mimicking therapeutics with minimal toxicity. The concept of context-dependent/specific CRABP1 signalosomes, which expands the repertoire of retinoid-modulated biological processes, enables the development of more dynamic and selective therapeutic strategies. Particularly, in translational applications, it is possible to maximize therapeutic efficacy and specificity by incorporating several elements for consideration, including a) CRABP1-dependency (only CRABP1-expressing cells will be affected), b) ligand-specificity, and c) signaling-pathway selectivity. In the future, this strategy may be further improved and expanded by considering the possibility of antagonistic modulators, allowing specific and rapid control of therapeutic duration, thus further minimizing potential toxicity. These potential improvements are possible because of the key concept of “signalosome”, which exploits the biological specificity of large molecular complexes that are more physiologically relevant. Therefore, drug design can not only be improved in meeting the needs for more efficacious therapeutic/pharmacological goals but can also be made more physiologically relevant.

## Figures and Tables

**Table 3:** Functions of IP-MS Proteins Implicated in Neurodegeneration.

GO Term	*Associated IP-MS Proteins	Implications in Neurodegeneration	Relevant Diseases	References
Actin filament binding (GO:0051015)	ACTR2, ACTR3, ACTR3B, AIF1L, ARPC3, ARPC4, ARPC5L, CAPZA2, CFL2, CORO1A, CORO1B, CTNNA1, EPB41L2, EZR, LIMA1, MSN, PLS1, RDX, SHTN1, TWF1, TWF2, WDR1, TMOD1	Dysregulation of actin dynamics or mutations in actin-binding proteins is associated with the following: Reduction in dendritic spines Impaired axonal transport and outgrowth Impaired membrane trafficking Synaptic dysfunction and loss Pathological rod-like inclusions consisting of actin and actin-binding proteins	AD, ALS, HD, PD	[56,57,58,59,60,61]
RNA binding (GO:0003723)	RPL30, RPL10, RPL31, RPL11, RPS27L, GSPT1, IGF2BP1, RACK1, RPL13, IGF2BP3, RPS11, HNRNPA0, CNBP, PABPC4, RPL22, RPS28, RPS29, SNRPG, PDCD4, RPS20, RPS21	Mutated, aggregated, or mis-localization of RNA-binding proteins is associated with the following: Dysregulated RNA metabolism, processing, and transport Altered translation of target mRNAs Formation of toxic RNA foci or cytoplasmic inclusions	AD, ALS, HD, PD, SMA	[62,63,64,65,66,67]
Ubiquitin-protein transferase regulator activity (GO:0055106)	RPL11, GLMN, PIN1, RPS20	Aberrant ubiquitin transferase expression, activity, or mutations have been associated with the following: Altered synaptic function and turnover Defective proteosome function Accumulation of disease-associated, mis-folded proteins (e.g., tau, mutant huntingtin, α-synuclein) Defective autophagy and mitophagy Disrupted mitochondrial homeostasis Defective cell-surface receptor and intracellular trafficking	AD, ALS, HD, PD	[68,69,70,71,72,73,74]
Protein kinase binding (GO:0019901)	PPP1CB, GLRX3, TWF2, RACK1, PIN1, MSN, SFN, EZR, PRKACA, HNRNPA0	Aberrant kinase expression, activity, or mutations have been associated with the following: Hyperphosphorylation of disease-related proteins (e.g., tau, mutant huntingtin, α-synuclein) Increased secretion of amyloid-β aggregates Altered cellular stress responses Impaired mitochondrial function and mitophagy Disrupted proteostasis Synaptic dysfunction Impaired axonal transport Enhanced neuronal death	AD, ALS, HD, PD	[75,76,77,78,79,80]
GTPase Activity (GO:0003924)	DRG1, RAB6B, GSPT1, SEPTIN11	Aberrant GTPase expression, activity, or mutations have been associated with the following: Altered cytoskeletal structure and dynamics Impaired mitochondrial function and mitophagy Increased ROS Altered cellular stress responses Dysregulated trafficking in axonal and dendritic compartments Defective membrane trafficking of cell-surface receptors Impaired synaptic transmission Imbalanced pro-survival versus death signaling	AD, ALS, HD, PD	[81,82,83,84,85,86,87,88,89]

*IP-MS protein are listed according to their official gene symbol.

## Data Availability

No new data were created or analyzed in this study.

## Abbreviations

The following abbreviations are used in this manuscript:

ACTR2	actin-related protein 2
ACTR3	actin-related protein 3
ACTR3B	actin-related protein 3B
AD	Alzheimer’s disease
AIF1L	allograft inflammatory factor-1-like
ALS	amyotrophic lateral sclerosis
APL	acute promyelocytic leukemia
ARPC3	actin-related protein 2/3 complex subunit 3
ARPC4	actin-related protein 2/3 complex subunit 4
ARPC5L	actin-related protein 2/3 complex subunit-5-like
CAPZA2	capping actin protein of muscle Z-line subunit alpha 2
CFL2	cofilin 2
CKO	CRABP1 knockout
CNBP	CCHC-type zinc finger nucleic-acid-binding protein
CNS	central nervous system
CORO1A	coronin 1A
CORO1B	coronin 1B
CRABP1	cellular retinoic-acid-binding protein 1
CTNNA1	catenin alpha 1
CYP450	cytochrome P450
DNA	deoxyribonucleic acid
DRG1	developmentally regulated GTP-binding protein 1
EIF2α	eukaryotic initiation factor 2 alphα
EPB41L2	erythrocyte membrane protein band 4.1-like-2
ERK1/2	extracellular signal-regulated kinases 1 and 2
EZR	ezrin
FDA	Food and Drug Administration
GCN2	general control nonderepressible 2
GLMN	glomulin
GLRX3	glutaredoxin 3
GO	gene ontology
GSPT1	G1 to S phase transition 1
GTP	guanosine-5’-triphosphate
HD	Huntington’s disease
HNRNPA0	heterogeneous nuclear ribonucleoprotein A0
HRI	heme-regulated inhibitor
IGF2BP1	insulin-like growth factor 2 mRNA-binding protein 1
IGF2BP3	insulin-like growth factor 2 mRNA-binding protein 3
IP-MS	immunoprecipitation-mass spectrometry
ISR	integrated stress response
LIMA1	LIM domain and actin-binding 1
LTP	long-term potentiation
MAPK	mitogen-activated protein kinase
MEK1/2	mitogen-activated protein kinase kinase 1 and 2
MN	motor neuron
MSN	moesin
NMJ	neuromuscular junction
NSC	neural stem cell
PABPC4	poly(A)-binding protein cytoplasmic 4
PD	Parkinson’s disease
PDCD4	programmed cell death 4
PERK	protein kinase R (PKR)-like endoplasmic reticulum kinase
PIN1	peptidylprolyl cis/trans isomerase, NIMA-interacting 1
PLS1	plastin 1
PPP1CB	protein phosphatase 1 catalytic subunit beta
PP2A	protein phosphatase 2
PRKACA	protein kinase cAMP-activated catalytic subunit alpha
atRA	all-trans retinoic acid
RAB6B	RAB6B, member RAS oncogene family
RACK1	receptor for activated C kinase 1
RAF-1	rapidly accelerated fibrosarcoma 1
RAS	rat sarcoma virus
RAR	retinoic-acid receptor
RDX	Radixin
RIP140	receptor-interacting protein 140
RNA	ribonucleic acid
ROS	reactive oxygen species
RPL	ribosomal protein large
RPS	ribosomal protein S
RXR	retinoid-X receptor
SAR	Structure–activity relationship
SEPTIN11	septin 11
SFN	Stratifin
SHTN1	shootin 1
SMA	spinal muscular atrophy
SNP	single-nucleotide polymorphism
SNRPG	small nuclear ribonucleoprotein polypeptide G
TMOD1	tropomodulin 1
TWF1	twinfilin actin-binding protein 1
TWF2	twinfilin actin-binding protein 2
UPR	unfolded protein response
WDR1	WD Repeat Domain 1
WT	wild-type
